# Complete Genome Sequence of Brucella abortus 2308, Isolated from an Abortion Storm on a Dairy Farm in India

**DOI:** 10.1128/mra.00550-22

**Published:** 2022-10-19

**Authors:** Amit Kumar, Malyaj R. Prajapati, Surendra Upadhyay, Anamika Bhordia, Vinod Kumar Singh, Jitender Singh, Anu Rahal, Ravindra Kumar, Anil Sirohi

**Affiliations:** a College of Biotechnology, Sardar Vallbhbhai Patel University of Agriculture & Technology, Meerut, Uttar Pradesh, India; b College of Veterinary Sciences, UP Pt. Deen Dayal Uphadhayay Pashu Chikitsa Vigyan Viswavidhyalay Evam Go-Anusandhan Sansthan, Mathura, Uttar Pradesh, India; c Division of Animal Health, ICAR—Central Institute for Research on Goats, Makhdoom, Farah, Mathura, Uttar Pradesh, India; University of Rochester School of Medicine and Dentistry

## Abstract

The present report communicates the first complete genome sequence of Brucella abortus strain 2308, isolated from an abortion storm on a dairy farm in India. Bacteria were isolated in pure culture from the placentas of aborted calves, and next-generation sequencing (NGS) revealed that the genome sequence length of the isolated strain is 3,285,606 bp, with a GC content of 57.25%, an *N*_50_ value of 296,426 bp, and an *L*_50_ value of 4, containing 3,119 coding DNA sequences (CDSs), 49 tRNAs, 1 transfer-messenger RNA (tmRNA), and 3 rRNA genes.

## ANNOUNCEMENT

Brucellosis is an zoonotic disease endemic to the Indian subcontinent and causes severe economic losses (1). There is no recommended treatment of brucellosis, and Brucella-positive animals are culled and sacrificed (2). However, in India, for religious reasons, Brucella-positive cattle are not sacrificed and are maintained in isolation (3). These infected animals might be a source of zoonotic infection (4). Brucella abortus 2308, the most pathogenic strain for humans (5), was not previously reported in India. Given the endemicity of brucellosis in the country (6), the involvement of Brucella abortus strain 2308 is of major concern. Meanwhile, the Government of India launched the National Animal Disease Control Programme (NADCP) in 2019 with the goal of eradicating brucellosis by 2030 using a Brucella abortus strain 19 vaccine. It is a live vaccine with long-lasting immunity and persistent antibodies (1). Thus, for successful vaccination, it is imperative to differentiate between infected and vaccinated animals (DIVA), in particularly in areas of endemicity like India where infected animals are not sacrificed. The availability of whole-genome sequencing of field isolates will be useful for developing a DIVA strategy. The present study reports the whole-genome sequence of Brucella abortus 2308, isolated from the placentas of aborted calves on a dairy farm located in Kanpur City, Uttar Pradesh, India. The aseptically collected placentas were sealed in a sterile collection bag, transported to the laboratory, and processed for isolation of the bacterium and its cultural, morphological, and biochemical characterization (7). The isolates were confirmed by amplification of the Brucella abortus *omp28* gene using previously described primers (8). For whole-genome sequencing, a single colony was picked for extraction of genomic DNA using the GeneJET genomic DNA purification kit (Thermo Fisher Scientific); genomic DNA libraries were then constructed using a NEBNext Ultra DNA library prep kit (Illumina, Inc., San Diego, CA). Each library was sequenced to produce 100-bp paired-end reads on an Illumina HiSeq 2000 instrument by Biokart India Pvt. Ltd. (Bengaluru, India), to achieve an average of 25× coverage per sample (range, 14× to 48×). The reads were processed using different software with default parameters unless otherwise specified. A total of 12,561,512 raw data reads were generated, with an average length of 149 nucleotides (nt), and a total of 12,560,987 clean reads were obtained, with an average length of 149 nt, using CLC Genomic Workbench version 7.0.4 (CLC bio, Qiagen, Denmark) to trim the Illumina adaptor sequences and remove low-quality reads. A total of 1,872,254,445 reads passed the quality filter; the reads averaged 152 bp long and had an average quality score above Q30 in more than 90% of the bases. The sequences were assembled using SPAdes version 3.11.0 (9) into 30 contigs at least 200 nt long, the largest contig comprising 609,417 bp, with a coverage of >10×, for a total of 3,278,307 bp, with a GC content of 57.25%, an *N*_50_ value of 296,426 bp, and an *L*_50_ value of 4. The genome contained 3,147 coding DNA sequences (CDSs), 48 contigs, 48 scaffolds, 30,901 open reading frames (ORFs), and 3,330 genes, with an average length of 853,828 nt, including 51 tRNAs, 1 transfer-messenger RNA (tmRNA), and 3 rRNA genes, as identified by annotation using the NCBI Prokaryotic Genome Annotation Pipeline (PGAP) version 4.11 (10) and RAST version 2.0 (11).

### Data availability.

This whole-genome shotgun project has been deposited at DDBJ/ENA/GenBank under the accession number JABBHV000000000 and the BioProject accession number PRJNA610713. The version described in this paper is version JABBHV000000000. The sequences have been submitted to the Sequence Read Archive (SRA) under the accession number SRR11526759.
